# DNA damage response pathway is a promising therapeutic target for extrachromosomal DNA‐positive tumours

**DOI:** 10.1002/ctm2.70519

**Published:** 2025-11-05

**Authors:** Congcong Tian, Haiyun Gan

**Affiliations:** ^1^ Guangdong Provincial Key Laboratory of Synthetic Genomics Key Laboratory of Quantitative Synthetic Biology Shenzhen Institute of Synthetic Biology Shenzhen Institute of Advanced Technology Chinese Academy of Sciences Shenzhen China

## 1 The characteristics of extrachromosomal DNA positive tumour

Circular extrachromosomal DNA (ecDNA) is acentric, mobile, large, and highly accessible circular DNA, which contains genes or regulatory regions. ecDNAs are present in nearly half of human cancers, but not in normal cells.^1^, ^2^ ecDNA content varies widely between tumour types and between individual tumours.^3^ Multiple ecDNA species and subspecies can co‐exist within a tumour population.^4^ Even in the same tumour cell, the sequencing and structure of ecDNA varies.^5^ As a result of acentric, ecDNA segregating asymmetrically to daughter cells leads to tumour heterogeneity and confers adaptation advantages to some of the tumour cells.^3^, ^4^ Different ecDNA species carrying oncogenes, enhancers, or immunoregulatory genes interact with each other and are coordinately inherited.^5^ The heterogeneity is further extended along with the dynamic evolution of ecDNA caused by structural rearrangement, DNA damage and recurrent mutations.^4^ Due to its ability to evade chemo‐, or target‐therapy and form an immune‐cold status, ecDNA in patient tumours drives tumour evolution, drug resistance, therapeutic escape, and leads to poor outcomes.^3^, ^6^

## 2 Drug resistance driven by extrachromosomal DNA

The formation, maintenance, amplification, integration and elimination of ecDNA are related to drug resistance of cancer cells. Double minutes (DM) with the *DHFR* gene are induced by higher doses and long duration of MTX treatment and promote MTX resistance in colon cancer HT29 cells.^7^ After withdrawal of the drug, DM with the *DHFR* gene disappeared accompanied with drug sensitivity restoration. Similarly, MAPK inhibitors can select ecDNAs carrying resistance genes in melanoma patients.^8^ The existence of ecDNA brings obstructs to target treatment as well. Attempts to target EGFR in glioblastoma (GBM) have had minimal success in comparison to other tumours. The failure is contributed by the variability of ecDNA. Target treatment with EGFR inhibitor erlotinib to GBM loading mice relieved most cancer initially accompanied by the copy number and expression of EGFRvIII ecDNA reduction.^9^ However, EGFR ecDNAs re‐emerged rapidly upon drug withdrawal, resulting in erlotinib resistance. Coincidentally, temporary removal of pemigatinib, which could reduce amplified *FGFR2* expression and *FGFR2* ecDNAs, resulted in recovery of *FGFR2* and its co‐segregated *MYC* ecDNA copy numbers.^5^ The complexity and variability of ecDNA restrict the therapeutic effects against specific elements within ecDNAs. Thus, identifying vulnerabilities generated by ecDNA is a critical priority for ecDNA‐directed oncology treatments.^3^

## 3 DNA damage response pathways are essential for **extrachromosomal** DNA maintenance

**FIGURE 1:**
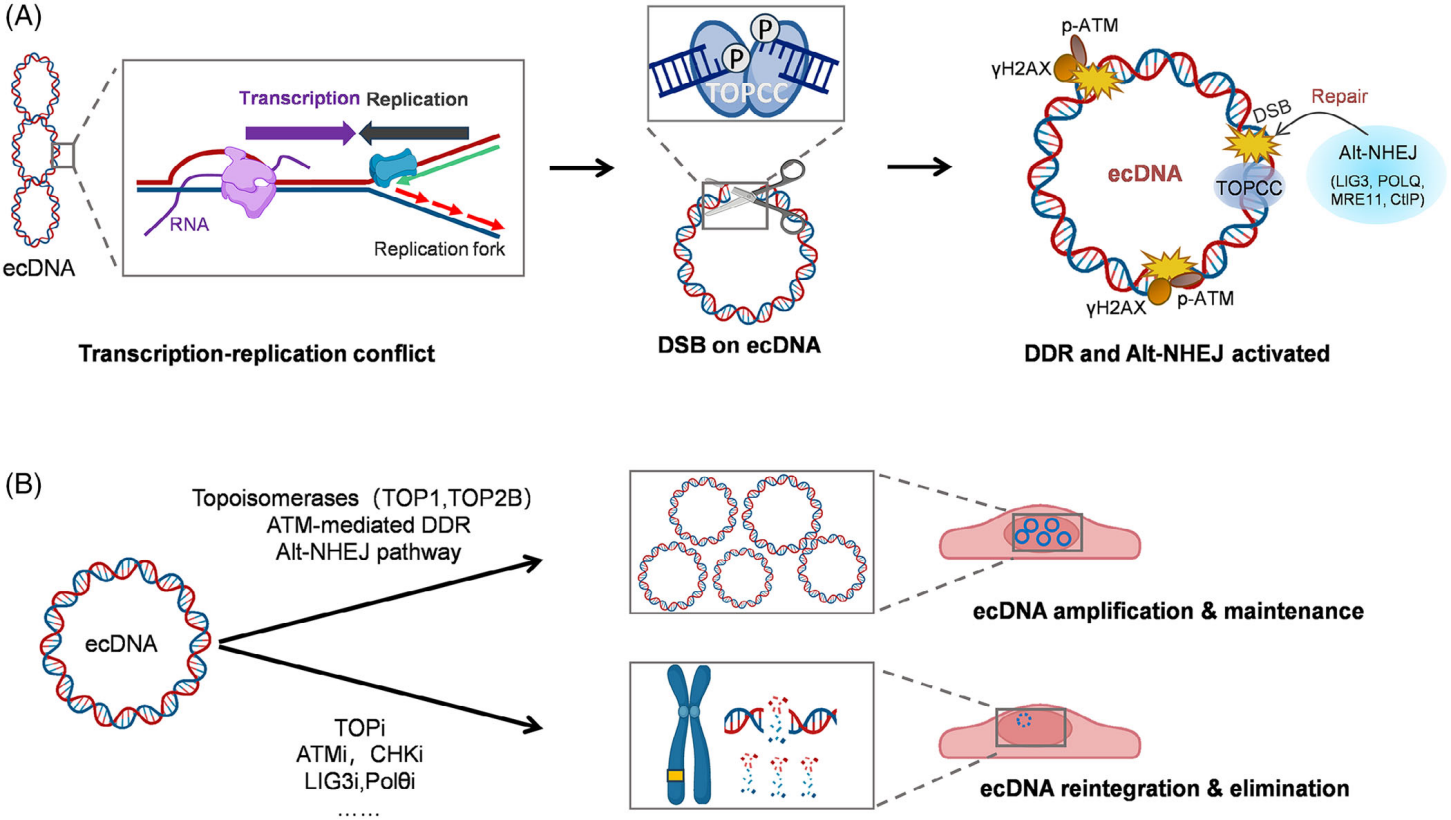
The DDR‐dependent maintenance mechanism of ecDNA and potential therapeutic targets for treating ecDNA+ tumours. (A) Transcription–replication conflicts are more frequent on ecDNA. In order to eliminate the topological stress produced by transcription and replication of ecDNA, topoisomerases cleaves one or two DNA strands, resulting in high level DSBs on ecDNA. DDR pathway, especially alt‐NHEJ, is activated, which safeguards the amplification and maintenance of ecDNA. (B) Topoisomerases (TOP1 and TOP2B), critical molecules in DDR pathway (ATM and CHK1/2) and especially key factors associated with alt‐NHEJ pathway (LIG3 and Polθ) are promising therapeutic targets for treating ecDNA+ tumours. ecDNA, extrachromosomal DNA; DDR, DNA damage response; alt‐NHEJ, alternative non‐homologous end joining.

ecDNAs are highly accessible chromatin with an altered gene‐regulatory architecture due to their circular structure, and are prone to form hubs that promote intermolecular cooperation, generating high‐level oncogenic transcription.^4^ ecDNAs replicate once per cell cycle and are stably maintained in ecDNA+ cells.^5^, ^10^ Transcription‐replication conflict arising from the high level of transcription from ecDNA induces replication stress on ecDNA.^10^, ^11^ Notably, proteins associated with nascent ecDNA are enriched in DNA repair pathways, indicating the important role of DNA damage response (DDR) in ecDNA maintenance and replication.^10^ Topoisomerases (TOP1 and TOP2B), which eliminate the topological stress during DNA transcription and DNA replication, are significant sources of DNA double‐strand breaks (DSBs) induced by ecDNA replication and are critical regulators of ecDNA‐induced DDR.^10^ LIG3 and other key factors associated with alternative non‐homologous end joining (alt‐NHEJ) pathway, rather than homologous recombination (HR) or NHEJ pathway, are crucial to the maintenance of ecDNA.^10^ Further cytotoxic analysis in ecDNA ± tumour cells reveals that ATM‐mediated DDR is a promising therapeutic target for treating ecDNA+ tumours. Knockdown or inhibiting TOP1 or TOP2B can decrease ecDNA levels and increase HSR signals notably. Using ATM inhibitor (ATMi) or checkpoint kinase protein inhibitor (CHKi) can successfully suppress the DDR pathway in ecDNA+ cells, and then the copy number of ecDNA is significantly reduced.^10^ Encouragingly, although the species of ecDNAs in various ecDNA+ cells are different, their sensitivity to drugs targeting ATM, CHK2, and TOP1 increased extensively.^10^ Similarly, due to the high replication stress, ecDNA+ cells are also highly sensitive to CHK1 inhibition.^11^ Shortly, ecDNA maintenance requires DDR, and inhibiting DDR impairs the circularization of ecDNA and promotes ecDNA+ cancer cell death. (See Figure 1).

## 4 The potential role of therapy‐induced DNA damage response on extrachromosomal DNA maintenance

It is worth noting that chemotherapy and radiotherapy lead to various forms of DNA damage and subsequently activation of diverse DDR pathways, which further promote radio‐resistance and chemo‐resistance.^12^, ^13^ Therapy‐induced activation of DDR pathways has a deeper biological significance in ecDNA+ tumour cells. DDR not only ensures genome replication and supports the cell cycle and division, but also is responsible for the maintenance and amplification of ecDNA, which provides more survival opportunities for cancer cells in a desperate situation. The degree of DNA damage in ecDNA+ cells is comparable to that therapy‐induced. However, ecDNA+ cells prefer the error‐prone alt‐NHEJ rather than HR or NHEJ to respond to the extensive DNA damage, making the repair process not limited by cell‐cycle phase and forming more subspecies of ecDNA, which further drives tumour evolution.

Multiple DDR inhibitors have been proven to be beneficial in the cancer treatment, either as monotherapy or in combination with chemotherapy and radiotherapy.^12^, ^13^ Targeting DDR processes could induce excessive DNA damages and enhance the efficacy of cytotoxic treatment which eventually leads to cancer cell death.^12^, ^13^ ATMi, CHK1/2i have been shown to sensitize cancer cells to chemotherapy and radiotherapy by impairing DNA repair, thereby enhancing treatment efficacy.^12^ TOP1 and TOP2 inhibitors are also frontline used in the treatment of solid tumours and haematological malignancies, by accumulation of torsional stress, triggering DDR pathways and apoptosis in rapidly proliferating cancer cells.^14^, ^15^ The combination of TOP1 inhibitors with DDR inhibitors could further enhance the anti‐tumour efficacy.^15^ Our results suggest that targeting ATM‐mediated DDR (especially, alt‐NHEJ) or topoisomerases (TOP1 and TOP2B) is far more therapeutic effective in ecDNA+ tumour cells. Differently, the treatment targeting DDR in ecDNA+ cancer cells decreased the DNA damage level instead of accumulating unrepaired DNA.^10^ The decrease of DNA damage signal is probably secondary to the integration and elimination of ecDNA rather than the direct effect of DDR targeting drugs. Patient selection is crucial for the clinical application of these inhibitors. Our results indicate that in the clinical trial of ATM, CHK1/2, TOP1/2B, LIG3 and Polθ inhibitors, it is worth considering including the tumour types with a high prevalence of ecDNA (such as liposarcomas, GBM, HER2^+^ breast cancer), which might produce unexpected therapeutic effects.

## 5 Therapeutic perspective of extrachromosomal DNA+ tumours

Considering the dynamic nature and heterogeneity of ecDNA, targeting the oncogene‐carrying ecDNA or their encoded proteins is challenging. However, ATM‐mediated DDR is essential for ecDNAs maintenance and replication, creating therapeutic vulnerabilities for ecDNA‐driven cancers. These targets provide opportunities to eliminate ecDNAs, regardless of their sources, species, or distributions in patient tumours. However, when a DDR inhibitor is used alone, the tendency of ecDNA to integrate or eliminate limits its application as monotherapy. Targeting DDR pathway could be given in combination with other targeted treatment, immunotherapy, chemotherapy or radiotherapy as a promising future direction.^10^

## AUTHOR CONTRIBUTIONS

Haiyun Gan conceived and designed the project. Congcong Tian wrote the manuscript and drew the schematic diagram.

## CONFLICT OF INTEREST STATEMENT

The authors declare no conflicts of interest.

## ETHICS STATEMENT

There are no ethical issues involved in this paper.
